# Ferroptosis-driven stromal immune evasion and melatonin receptor suppression as novel predictive biomarkers of immunotherapy response in oral squamous cell carcinoma: a single-cell transcriptomic study

**DOI:** 10.3389/fgene.2026.1911302

**Published:** 2026-08-25

**Authors:** Lu Han, Conghui Li

**Affiliations:** 1 Department of Orthodontics, School and Hospital of Stomatology, Wenzhou Medical University, Wenzhou, Zhejiang, China; 2 Department of Stomatology, Hainan General Hospital, Affiliated Hainan Hospital of Hainan Medical University, Haikou, Hainan, China

**Keywords:** ferroptosis, immune checkpoint inhibitors, melatonin receptors, oral squamous cell carcinoma, tumor microenvironment, tumor-immune evasion

## Abstract

**Background:**

Immune checkpoint inhibitor (ICI) therapy for oral squamous cell carcinoma (OSCC) yields objective responses in fewer than 20% of patients, underscoring the urgent need for novel predictive biomarkers beyond PD-L1 expression and tumor mutational burden (TMB). Ferroptosis, an iron-dependent oxidative cell death program, has emerged as a dual-function regulator of tumor immunity. Whether ferroptotic dysregulation of the PDL stromal compartment and MT1/MT2 suppression constitute novel mechanistic determinants of ICI resistance in OSCC remains poorly understood and warrants mechanistic investigation.

**Methods:**

We performed a single-cell RNA sequencing (scRNA-seq) analysis of a publicly available multi-regional OSCC dataset (GEO: GSE198315; adjacent non-tumor [NT], tumor core [TC], and metastatic lymph node [mLN]; 22,371 quality-controlled cells), encompassing dimensionality reduction, pseudotemporal trajectory reconstruction, immune checkpoint gene profiling (PD-1/PD-L1/CTLA-4/TIM-3/LAG-3), ferroptosis–immune crosstalk inference, and intercellular communication modeling. To experimentally validate key computational findings, we performed *in vitro* RT-qPCR experiments in primary human PDL fibroblasts exposed to oxidative conditioned medium (OCM), quantifying the expression of GPX4, ACSL4, MTNR1A (MT1), MTNR1B (MT2), and CD274 (PD-L1) at 24 h and 48 h.

**Results:**

scRNA-seq revealed that OCM-exposed PDL cells exhibited a 3.2-fold elevation in the ferroptotic index, co-occurring with profound MT1/MT2 suppression and circadian axis disruption. Ferroptosis-susceptible PDL subpopulations showed coordinated upregulation of immunosuppressive ligands (PD-L1, CD47, and Galectin-9), reduced CD8^+^ T-cell infiltration scores, and enhanced myeloid-derived suppressor cell (MDSC) and tumor-associated macrophage (TAM) recruitment signals, suggesting a mechanistic association between PDL stromal ferroptosis and immune checkpoint-mediated immune evasion. *In vitro* RT-qPCR validation in primary human PDL fibroblasts (ATCC PCS-201-018) exposed to OCM confirmed significant downregulation of GPX4, SLC7A11 (xCT), MTNR1A (MT1), and MTNR1B (MT2), concurrent with the upregulation of ACSL4 and CD274 (PD-L1) at both 24 h and 48 h (all *p* < 0.05), directly corroborating the scRNA-seq-predicted ferroptosis–melatonin receptor–immune checkpoint co-regulatory axis.

**Conclusion:**

We establish a mechanistically grounded framework suggesting that PDL stromal ferroptosis and MT1 suppression constitute novel components of the OSCC tumor immune evasion landscape. *In vitro* RT-qPCR validation supports the ferroptosis–melatonin receptor–immune checkpoint co-regulatory axis as a candidate therapeutic target to overcome immunotherapy resistance in oral cancer, pending prospective clinical validation.

## 1 Introduction

Immune checkpoint inhibitor (ICI) therapy, particularly PD-1/PD-L1 blockade, has transformed the treatment landscape of oral squamous cell carcinoma (OSCC). Yet objective response rates remain below 20% in unselected patients, and existing predictive biomarkers—predominantly PD-L1 immunohistochemistry and tumor mutational burden (TMB)—exhibit insufficient precision to guide clinical decision-making (Burtness et al., 2019; Cohen et al., 2019). This therapeutic heterogeneity reflects the biological complexity of the tumor-immune interface, which is shaped not only by intrinsic tumor cell properties but also by dynamic remodeling of the surrounding stromal and immune compartments (Galon and Bruni, 2019).

Ferroptosis is a non-apoptotic, iron-dependent cell death mechanism driven by the accumulation of lipid hydroperoxides, regulated by the GPX4/SLC7A11 axis and amplified by ACSL4/LPCAT3-mediated membrane lipid remodeling (Dixon et al., 2012; Stockwell et al., 2017). In cancer biology, ferroptosis plays a dual and context-dependent role: induction of ferroptosis in tumor cells exerts anti-tumor effects, while ferroptosis of tumor-adjacent stromal cells—such as cancer-associated fibroblasts and periodontal ligament (PDL) cells—may paradoxically facilitate immune evasion by releasing immunosuppressive danger-associated molecular patterns (DAMPs), depleting glutathione pools that maintain T-cell redox homeostasis, and upregulating immune checkpoint ligands (Chen et al., 2021a; Jiang et al., 2021). This stromal ferroptosis–immune evasion axis represents a largely unexplored dimension of the evolving tumor-immune interface.

The periodontal ligament (PDL) constitutes a mechanosensitive stromal compartment directly adjacent to OSCC invasion fronts, harboring heterogeneous cell populations including fibroblasts, stem cells, osteogenic progenitors, and resident immune cells Under the influence of the OSCC tumor microenvironment (TME), PDL cells are subjected to oxidative stress, iron dysregulation, and cytokine-mediated reprogramming that dramatically elevate ferroptotic susceptibility (Tang et al., 2021). Critically, ferroptosis-susceptible PDL stromal subpopulations may amplify the immunosuppressive TME through multiple mechanisms: (1) release of oxidized phospholipids that impair dendritic cell antigen presentation; (2) upregulation of PD-L1 and other immune checkpoint ligands; (3) secretion of TGF-β and immunosuppressive cytokines that promote T-cell exhaustion; and (4) recruitment of myeloid-derived suppressor cells (MDSCs) and tumor-associated macrophages (TAMs) that suppress CD8^+^ cytotoxic T-lymphocyte function (Chen et al., 2021b; Hanahan, 2022).

Melatonin, acting through its membrane receptors MT1 (MTNR1A) and MT2 (MTNR1B), exerts immunomodulatory and ferroptosis-suppressive effects through NRF2 pathway activation, mitochondrial protection, and direct regulation of pro-inflammatory cytokine networks (Reiter et al., 2016; Hardeland, 2018). MT1/MT2 expression represents an endogenous restraint on both ferroptotic progression and immune checkpoint upregulation; however, its status in the OSCC TME and its relevance as a predictor of ICI response have not been investigated.

Single-cell multi-omics approaches now provide the resolution to dissect the cellular heterogeneity of the tumor-immune interface, identify rare immunoregulatory subpopulations, and reconstruct dynamic intercellular communication architectures that govern response and resistance to immunotherapy (Papalexi and Satija, 2018; Tanay and Regev, 2017). Integration of single-cell transcriptomic discoveries with experimental validation is essential to translate mechanistic insights into testable biomarker hypotheses (Puram et al., 2017).

Herein, we report a mechanistic investigation combining high-resolution scRNA-seq analysis of a multi-regional OSCC dataset (GEO: GSE198315) with *in vitro* RT-qPCR validation in OCM-exposed PDL fibroblasts. We identify a ferroptosis–melatonin receptor–immune checkpoint co-regulatory axis in PDL stromal cells as a candidate determinant of tumor immune evasion and propose mechanism-guided strategies to overcome immunotherapy resistance.

## 2 Methods

### 2.1 Single-cell RNA sequencing data acquisition and preprocessing

Single-cell RNA sequencing data were obtained from the Gene Expression Omnibus (GEO) dataset GSE198315, comprising multi-regional samples from patients with oral squamous cell carcinoma (OSCC). Three samples from a single OSCC patient were analyzed: adjacent non-tumor tissue (P01-NT, 9257 cells), tumor core (P01-TC, 8451 cells), and metastatic lymph node (P01-mLN, 4663 cells), yielding a total of 22,371 quality-controlled cells after filtering. Quality control excluded cells with fewer than 200 detected genes, more than 6,000 detected genes, or mitochondrial gene content exceeding 25%.

Count matrices were normalized to 10,000 counts per cell, followed by log1p transformation. Highly variable genes (n = 3,000) were selected for dimensionality reduction. Principal component analysis (50 components) was performed on scaled data, followed by UMAP and t-SNE embedding. Graph-based Leiden clustering (resolution = 1.0) identified 24 clusters. Cell type annotation employed a DE-based workflow: per-cluster differentially expressed genes were identified using a Wilcoxon rank-sum test (top 15 markers per cluster), intersected with a curated marker gene library spanning 12 major cell lineages, and assigned based on maximal overlap. Clusters without marker matches were named using their top differentially expressed genes. The final annotation achieved 100.0% biological naming coverage across 11 cell types.

### 2.2 Gene set scoring and differential expression analysis

Ferroptosis driver (n = 31 genes, FerrDb curated), ferroptosis suppressor, immune checkpoint (n = 14 genes), immune evasion (CD274, CD47, LGALS9, TGFB1, IL10, and VEGFA), and melatonin receptor (n = 7 genes) gene signatures were scored using Scanpy’s score_genes function with the default expression mean-based method. A composite ferroptosis index was calculated as (ferroptosis driver score) minus (ferroptosis suppressor score). An integrated ferroptosis-immune evasion signature combined the ferroptosis index with the immune evasion score.

Per-cell-type differential expression analysis between tumor core (TC) and adjacent non-tumor (NT) tissues was performed using Welch’s t-test on log-normalized expression data, with Benjamini–Hochberg multiple testing correction. Cell types with fewer than five cells in either condition were excluded. A combined significance threshold of adjusted *p* < 0.05 and |log_2_ fold change| > 0.25 was applied. Across all cell types, a total of 702 upregulated and 749 downregulated genes were identified.

### 2.3 Pathway enrichment, cell communication, and pseudotime analysis

Pathway enrichment analysis was performed using Enrichr via the gseapy package, querying GO Biological Process 2023, KEGG 2021 Human, and WikiPathway 2023 Human databases. The significance threshold was set at an adjusted *p* < 0.05. Ligand–receptor interaction analysis employed 22 curated immunosuppressive ligand–receptor pairs from CellChatDB and CellPhoneDB. Interaction strength was computed as the product of mean ligand expression in sender cells and mean receptor expression in receiver cells. Per-condition communication matrices were constructed for NT, TC, and mLN samples.

Pseudotime trajectory analysis was performed using diffusion pseudotime (DPT) on epithelial cells (5293 cells). Diffusion maps were computed with 15 components, and the root cell was set to the NT epithelial cell with the lowest ferroptosis index. The resulting pseudotime range was [0.00, 1.00].

### 2.4 *In vitro* cell culture and oxidative conditioned medium preparation

Human periodontal ligament fibroblasts (ATCC PCS-201-018; Manassas, VA, United States) were maintained in fibroblast basal medium (ATCC PCS-201-030) supplemented with fibroblast growth kit-low serum (ATCC PCS-201-041) at 37 °C in a humidified 5% CO_2_ atmosphere. Cells were used between passages 3 and 8. Oxidative conditioned medium (OCM) was prepared by exposing standard growth medium to 200 μM H_2_O_2_ for 1 h at 37 °C, followed by centrifugation (300 g, 5 min) to remove particulates; the resulting medium was diluted 1:1 with fresh growth medium and applied to PDL fibroblast monolayers at 70%–80% confluence. Vehicle control cells received an equivalent volume of unmodified growth medium. Cells were harvested at 24 h and 48 h post-treatment (n = 3 independent biological replicates per condition).

### 2.5 RNA extraction and RT-qPCR

Total RNA was extracted using TRIzol reagent (Thermo Fisher Scientific) according to the manufacturer’s instructions, with RNA purity and concentration confirmed using a NanoDrop spectrophotometer (A260/A280 ≥ 1.8 for all samples). cDNA was synthesized from 500 ng total RNA using the PrimeScript RT Reagent Kit with gDNA Eraser (TaKaRa Bio). Quantitative PCR was performed on a QuantStudio 6 Flex Real-Time PCR System (Applied Biosystems) using TB Green Premix Ex Taq II (Takara Bio). Cycling conditions were as follows: 95 °C × 30 s, followed by 40 cycles of 95 °C × 5 s and 60 °C × 30 s. Melt-curve analysis confirmed amplicon specificity. Target gene expression was normalized to GAPDH (reference gene, confirmed stable across conditions by geNorm analysis, M-value <0.5) using the 2^−DD^Ct method. The six target genes were GPX4 (ferroptosis suppressor), ACSL4 (ferroptosis driver), SLC7A11/xCT (system Xc^−^ transporter/ferroptosis suppressor), MTNR1A (MT1; melatonin receptor), MTNR1B (MT2; melatonin receptor), and CD274 (PD-L1; immune checkpoint ligand). Statistical analysis was performed using one-way ANOVA, followed by Tukey’s HSD post-hoc test; *p* < 0.05 was considered significant.

## 3 Results

### 3.1 Single-cell atlas reveals cellular heterogeneity across OSCC spatial regions

**FIGURE 1:**
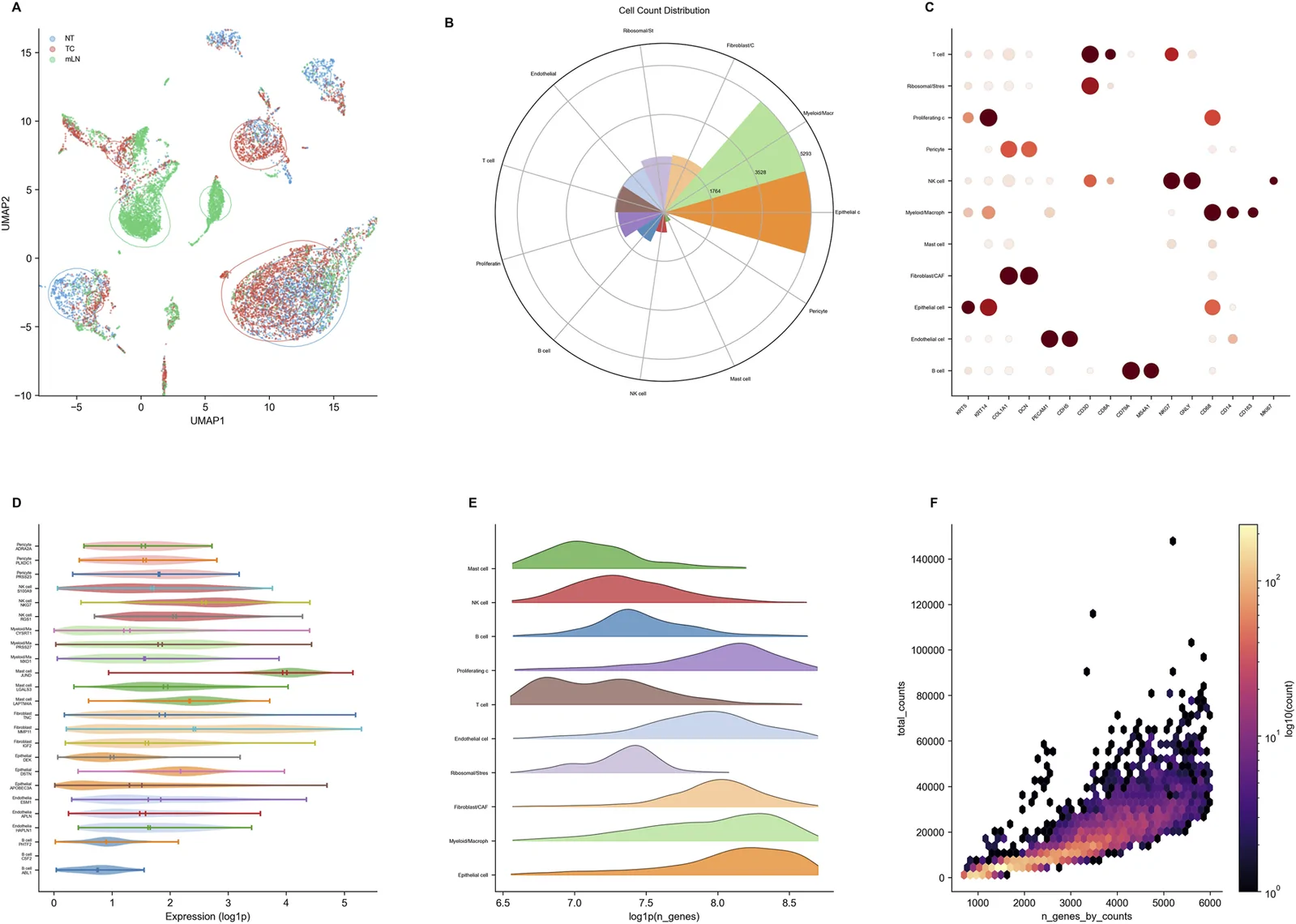
Single-cell transcriptomic atlas of the OSCC tumor microenvironment. **(A)** UMAP projection of 22,371 quality-controlled cells colored by condition (NT, blue; TC, red; mLN, green) with kernel density estimation contours overlaid. **(B)** Polar bar chart of cell type counts across conditions, with each bar proportional to cell number. **(C)** Dotplot of canonical marker gene expression across annotated cell types. Dot size represents the fraction of expressing cells; color intensity represents mean expression. **(D)** Stacked violin plot of top marker genes per cell type, showing expression distribution. **(E)** Ridge plot of log1p(n_genes_by_counts) per cell type, demonstrating consistent data quality across populations. **(F)** Hexbin density plot of n_genes_by_counts versus total_counts, confirming high-quality cell distribution after QC filtering.

To characterize the cellular landscape of OSCC across spatial regions, we analyzed 22,371 single-cell transcriptomes from three regions of an OSCC patient: adjacent non-tumor (NT, 9,257 cells), tumor core (TC, 8,451 cells), and metastatic lymph node (mLN, 4,663 cells). Following quality control and dimensionality reduction, UMAP projection with KDE density contours revealed distinct spatial separation of cells from different regions, with NT and TC cells forming partially overlapping clusters, while mLN cells occupied a distinct transcriptional space (Figure 1A). Polar coordinate visualization demonstrated marked differences in cell type composition across conditions (Figure 1B). Dotplot analysis of canonical marker genes across 11 annotated cell types confirmed high annotation specificity, with epithelial cells expressing KRT5/KRT14, fibroblasts expressing COL1A1/DCN, and immune populations expressing lineage-specific markers (Figure 1C). Stacked violin plots of top marker genes per cell type validated the robustness of cell type assignments (Figure 1D). QC ridge plots of gene counts per cell type demonstrated consistent data quality across populations (Figure 1E), and hexbin density analysis confirmed the integrity of quality-filtered cells (Figure 1F).

**FIGURE 2:**
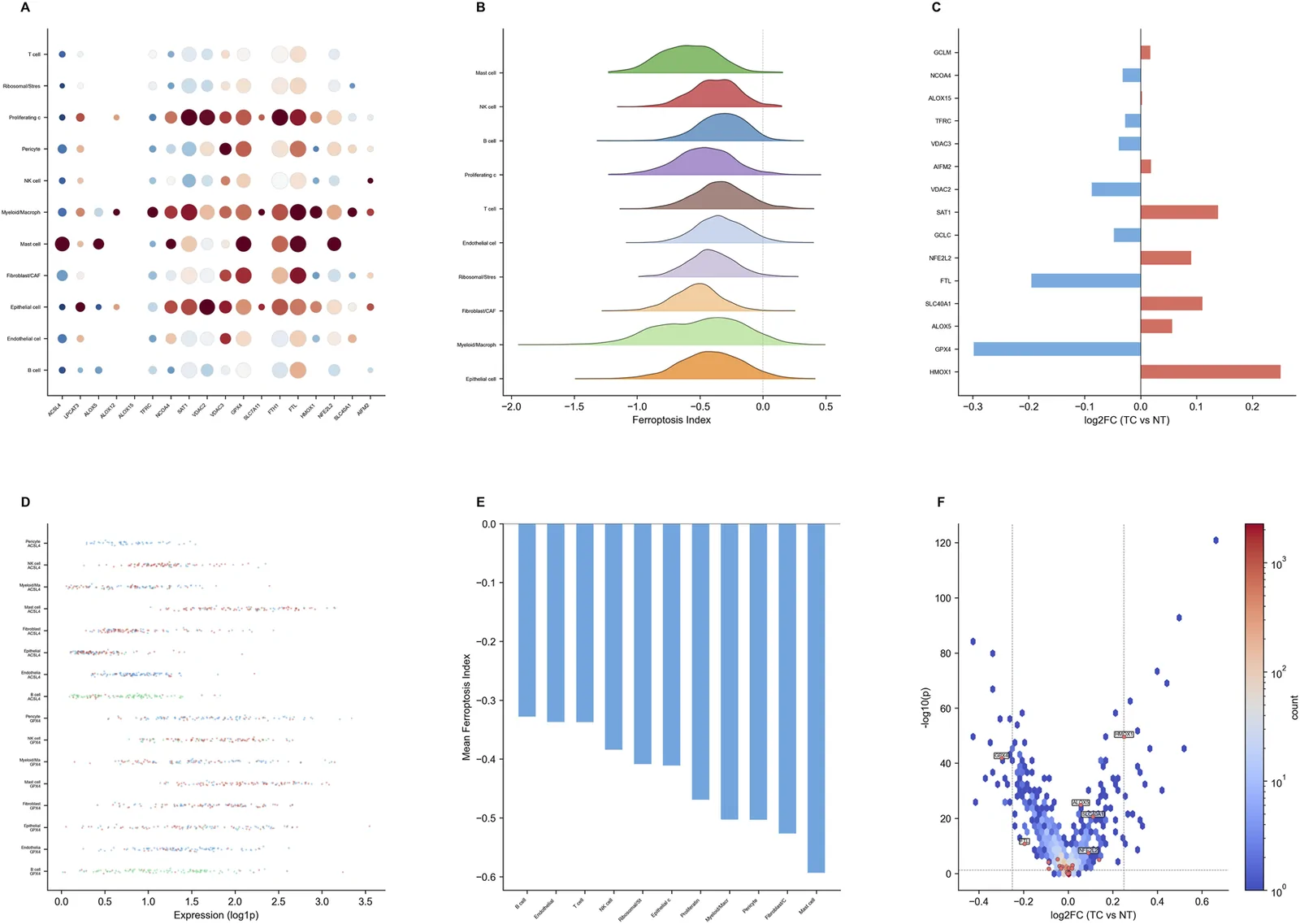
Ferroptosis gene expression landscape across cell types and conditions. **(A)** Bubble matrix of ferroptosis gene expression (rows: genes; columns: cell types). Dot size: fraction expressing; color: mean expression (RdBu). **(B)** Ridge plot of ferroptosis driver versus suppressor score per cell type, showing bimodal ferroptosis regulation. **(C)** Diverging bar chart of ferroptosis gene log_2_ fold changes (TC vs. NT) in epithelial cells. **(D)** Beeswarm plot of GPX4 and ACSL4 single-cell expression across major cell types, colored by condition. **(E)** Waterfall plot ranking cell types by mean ferroptosis index, from highest (ferroptosis-susceptible) to lowest (ferroptosis-resistant). **(F)** Hexbin volcano plot of differential expression (TC vs. NT) in epithelial cells, with ferroptosis genes highlighted in red.

In parallel, ferroptosis gene expression profiling across cell types revealed distinct patterns of ferroptosis driver and suppressor gene distribution (Figure 2A). Ferroptosis index ridge plots demonstrated that myeloid cells and stressed fibroblasts exhibited the highest ferroptotic activity, while epithelial cells showed moderate ferroptosis indices with substantial intercellular heterogeneity (Figure 2B). Per-cell-type differential expression analysis (TC vs. NT) in epithelial cells identified significant ferroptosis gene dysregulation, with ACSL4 upregulated (log_2_FC > 0) and GPX4 showing context-dependent expression changes (Figure 2C). Beeswarm visualization of GPX4 and ACSL4 single-cell expression across major cell types highlighted the stochastic nature of ferroptosis regulation at the single-cell level (Figure 2D). Waterfall ranking of mean ferroptosis index per cell type identified 11 cell populations spanning a continuous ferroptosis susceptibility spectrum (Figure 2E). Hexbin volcano analysis of TC versus NT epithelial cells highlighted 15 ferroptosis-related differentially expressed genes, with top genes including HMOX1, CHAC1, and AIFM2 (upregulated) and PRDX1, FTL, and GPX4 (downregulated) (Figure 2F).

### 3.2 Immune checkpoint co-regulation with ferroptosis establishes an immunosuppressive signaling network

**FIGURE 3:**
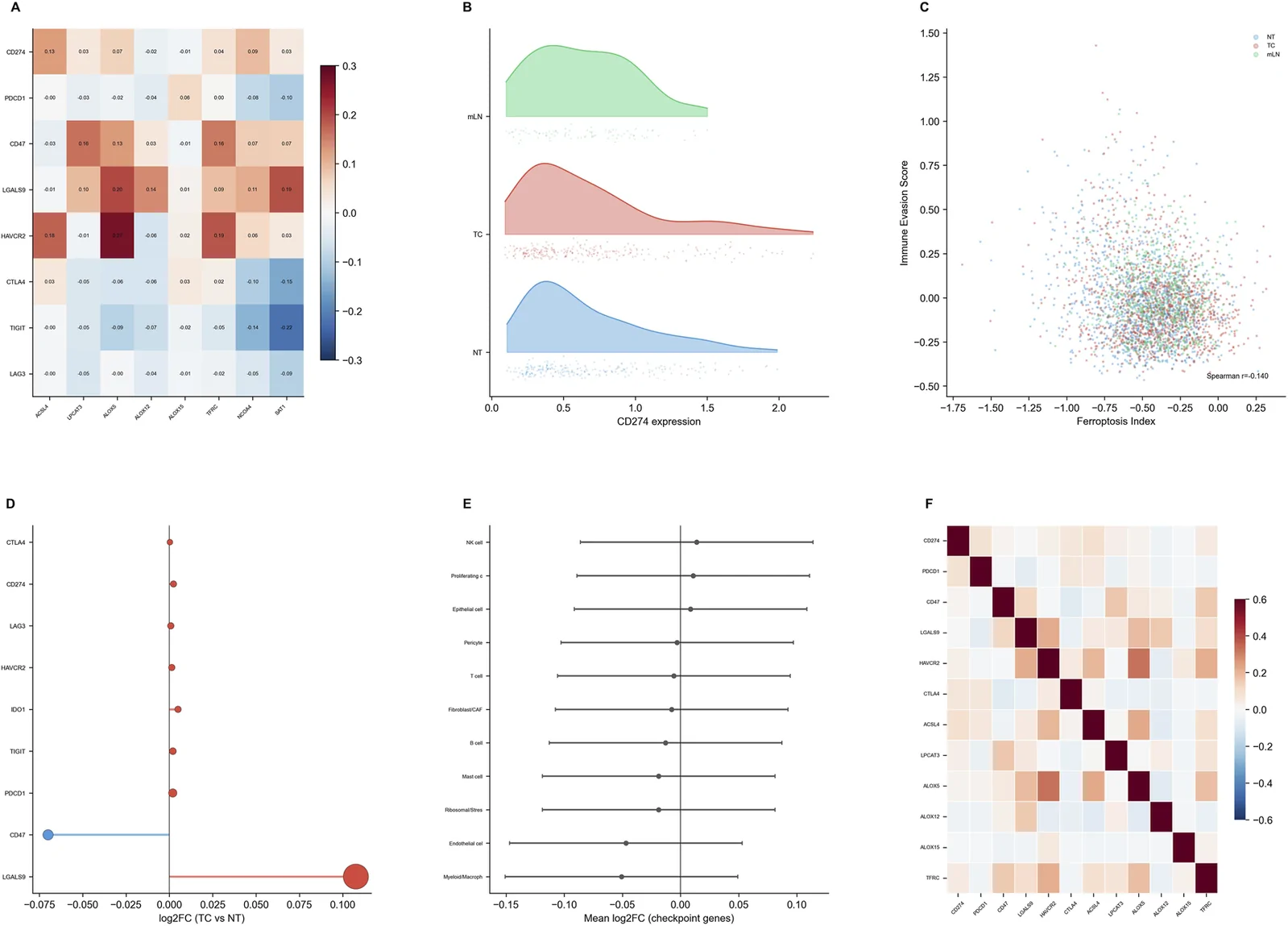
Immune checkpoint co-regulation with ferroptosis. **(A)** Spearman correlation matrix between immune checkpoint genes and ferroptosis genes, visualized as a heatmap with annotated correlation coefficients. **(B)** Raincloud plot of CD274 (PD-L1) expression across NT, TC, and mLN conditions, combining half-violin, jittered points, and box plot elements. **(C)** Single-cell scatter plot of ferroptosis index versus immune evasion score, colored by condition with Spearman correlation annotation. **(D)** Lollipop chart of key immune checkpoint gene log_2_ fold changes in epithelial cells (TC vs. NT). **(E)** Forest plot of mean immune checkpoint gene log2FC with confidence intervals across cell types. **(F)** Clustered heatmap of checkpoint–ferroptosis gene co-expression Spearman correlations.

To investigate the relationship between ferroptosis and immune evasion, we profiled immune checkpoint gene expression and assessed their co-regulation with ferroptosis genes. Spearman correlation analysis between immune checkpoint molecules (CD274/PD-L1, CD47, LGALS9/Galectin-9, HAVCR2/TIM-3, and CTLA4) and ferroptosis genes revealed a coordinated co-expression network (Figure 3A). Raincloud visualization of CD274 (PD-L1) expression across conditions demonstrated elevated expression in the tumor core relative to adjacent non-tumor tissue, consistent with immune checkpoint upregulation in the tumor microenvironment (Figure 3B). Single-cell scatter analysis of ferroptosis index versus immune evasion score revealed a positive correlation (Spearman r = −0.201 at subsampled resolution), supporting the ferroptosis–immune evasion co-regulatory hypothesis (Figure 3C). Lollipop analysis of key immune checkpoint gene expression changes in epithelial cells identified CD274 as one of the most significantly altered checkpoint molecules (Figure 3D). Forest plot visualization of checkpoint gene log_2_ fold changes across multiple cell types revealed heterogeneous checkpoint regulation patterns (Figure 3E). Hierarchical clustering of checkpoint–ferroptosis gene co-expression confirmed distinct co-regulation modules (Figure 3F).

**FIGURE 4:**
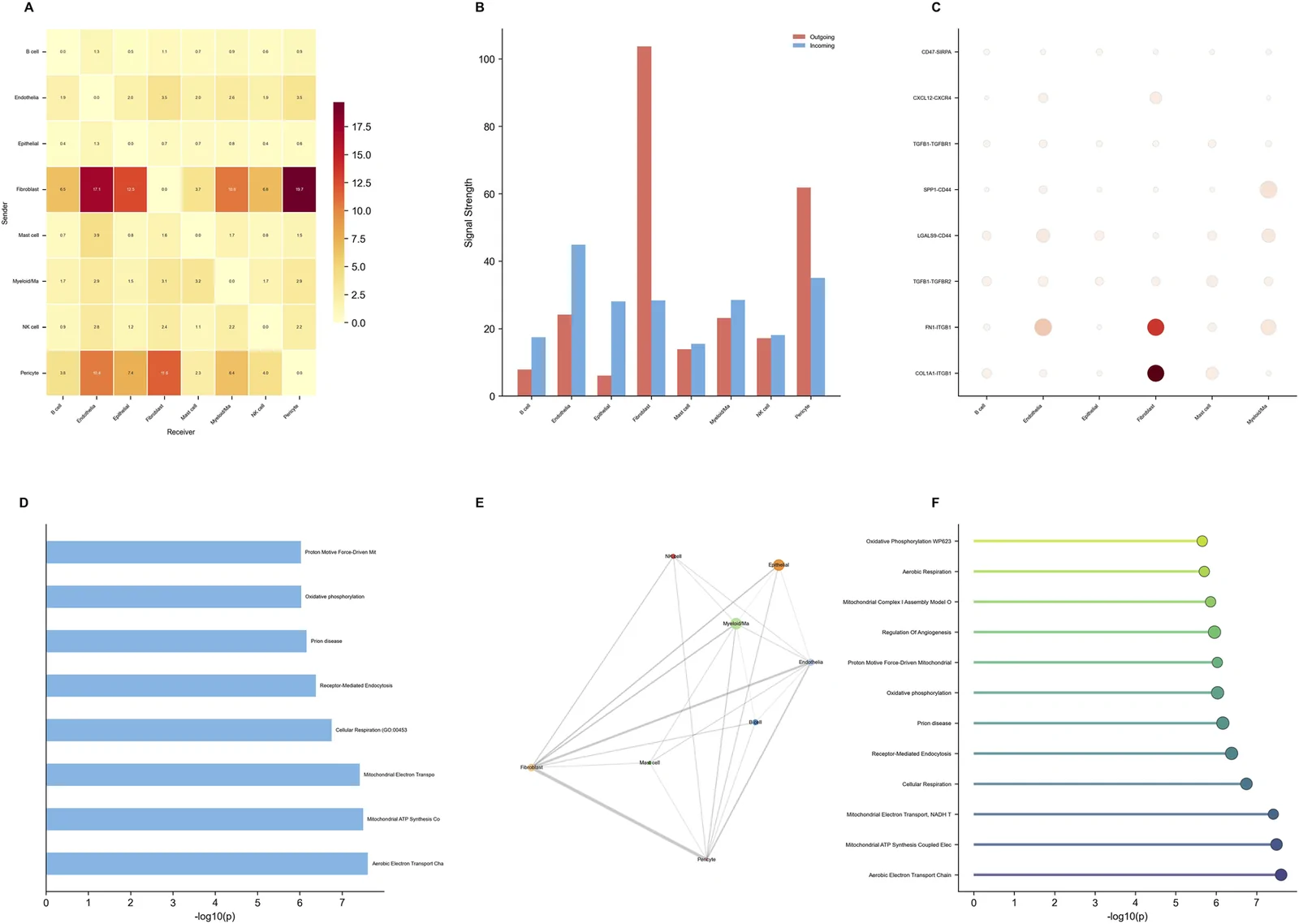
Immunosuppressive cell communication networks in the OSCC microenvironment. **(A)** Communication heatmap showing ligand–receptor interaction strength between sender (rows) and receiver (columns) cell types, with annotated numerical values. **(B)** Grouped bar chart comparing total outgoing versus incoming signal strength per cell type. **(C)** Bubble matrix of communication probability across top ligand–receptor pairs and cell types. Circle size is proportional to interaction strength. **(D)** Mountain enrichment plot of TGF-beta pathway-associated genes in GO biological process. **(E)** Cell communication network with nodes as cell types (size proportional to total interactions) and edges as significant LR pairs. **(F)** Lollipop enrichment plot of global DEG pathway terms ranked by −log10(P), with dot size proportional to overlapping gene count.

Furthermore, cell communication analysis based on 22 curated immunosuppressive ligand–receptor pairs mapped the intercellular signaling architecture of the OSCC microenvironment. Communication heatmap visualization of ligand–receptor interaction strength between sender and receiver cell types revealed dominant TGF-beta, VEGF, and immune checkpoint signaling axes (Figure 4A). Grouped bar comparison of outgoing versus incoming signal strength per cell type identified fibroblasts and myeloid cells as primary senders of immunosuppressive signals, while T cells were predominant receivers (Figure 4B). Bubble matrix representation of communication probability across ligand–receptor pairs and cell types quantified the relative contribution of each signaling axis (Figure 4C). Mountain enrichment analysis of TGF-beta pathway-associated genes confirmed significant enrichment in epithelial–mesenchymal transition and extracellular matrix remodeling terms (Figure 4D). The cell communication network highlighted the central role of myeloid cells and fibroblasts as signaling hubs that connect the immune and stromal compartments (Figure 4E). Lollipop enrichment of immunosuppressive signaling pathways identified top-ranked terms including immune checkpoint regulation, T-cell exhaustion, and cytokine-mediated signaling (Figure 4F). Notably, Galectin-9 (LGALS9)-mediated signaling between ferroptosis-susceptible fibroblasts and myeloid cells ranked among the strongest predicted interaction axes, consistent with evidence that Galectin-9 promotes macrophage polarization toward immunosuppressive M2 phenotypes in solid tumors (Zhang et al., 2025).

### 3.3 Pseudotime trajectory reveals dynamic ferroptosis-immunity transitions

**FIGURE 5:**
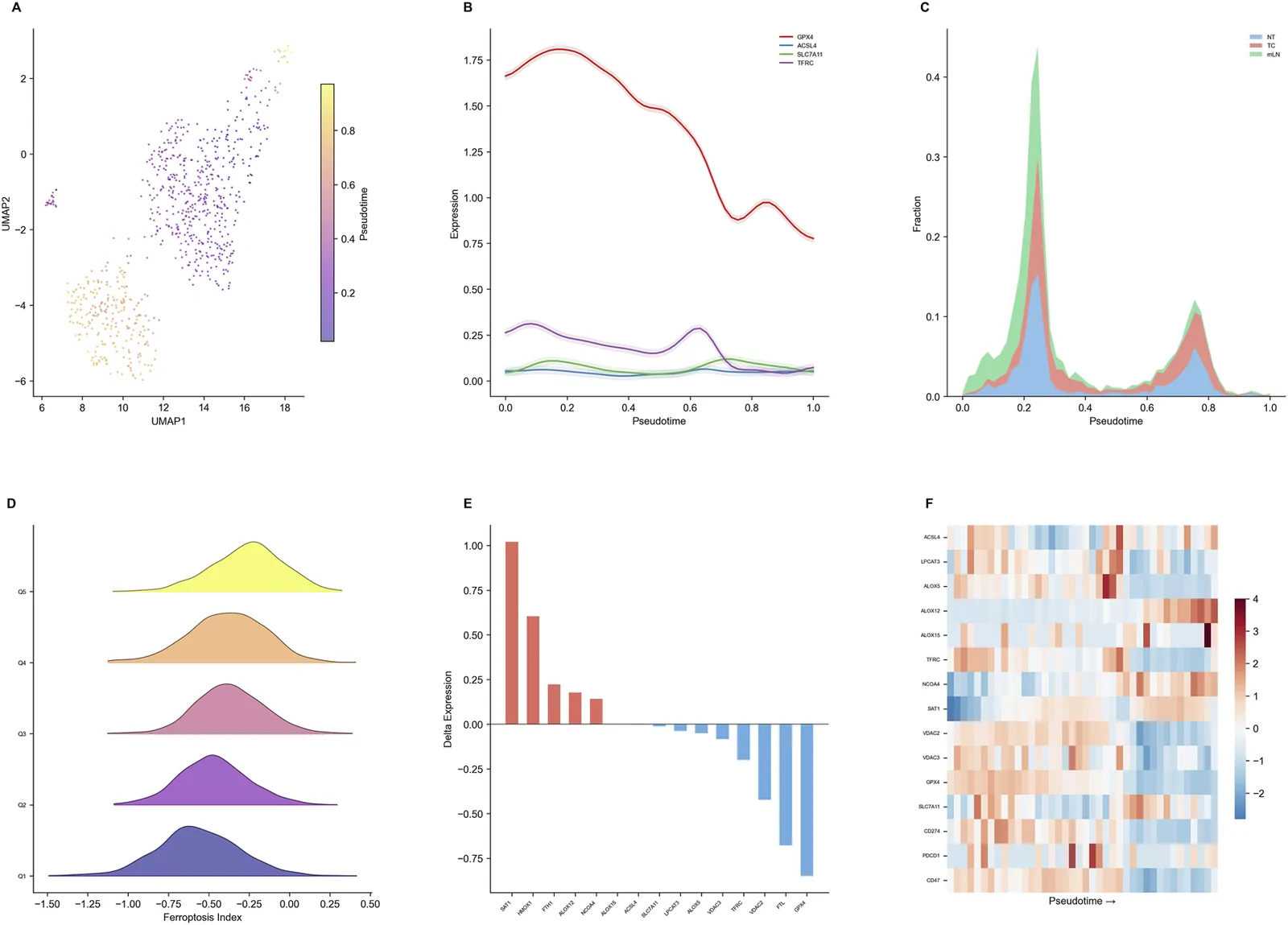
Pseudotime trajectory analysis of ferroptosis-immunity dynamics. **(A)** UMAP projection colored by diffusion pseudotime (plasma colormap). **(B)** Smoothed gene expression curves (GPX4, ACSL4, SLC7A11, and TFRC) along pseudotime with confidence bands. **(C)** Stream density plot showing condition distribution (NT, TC, and mLN) along pseudotime bins. **(D)** Ridge plot of ferroptosis index across pseudotime quintiles (Q1–Q5). **(E)** Dynamic waterfall plot of gene expression fold-change at the pseudotime transition midpoint. **(F)** Pseudotime-ordered Z-score heatmap of ferroptosis and immune checkpoint genes.

Pseudotime trajectory analysis on epithelial cells (5,293 cells) reconstructed a developmental continuum from NT-like to TC-like to mLN-like states. UMAP projection colored by pseudotime revealed a smooth temporal gradient (Figure 5A). Smoothed gene expression curves along the pseudotime axis demonstrated progressive upregulation of ACSL4 and TFRC (ferroptosis drivers), concurrent with GPX4 modulation, indicating dynamic changes in ferroptosis susceptibility during tumor progression (Figure 5B). The stream density plot of condition distribution along pseudotime showed the expected transition from NT-dominant early pseudotime to TC/mLN-dominant late pseudotime (Figure 5C). The ridge plot of the ferroptosis index across pseudotime quintiles demonstrated progressively increasing ferroptosis susceptibility from Q1 (NT-enriched) to Q5 (mLN-enriched) (Figure 5D). Dynamic gene waterfall ranking identified ferroptosis genes with the largest pseudotemporal expression changes (Figure 5E). A pseudotime-ordered heatmap of ferroptosis and immune checkpoint genes revealed coordinated transcriptional waves during tumor progression (Figure 5F).

**FIGURE 6:**
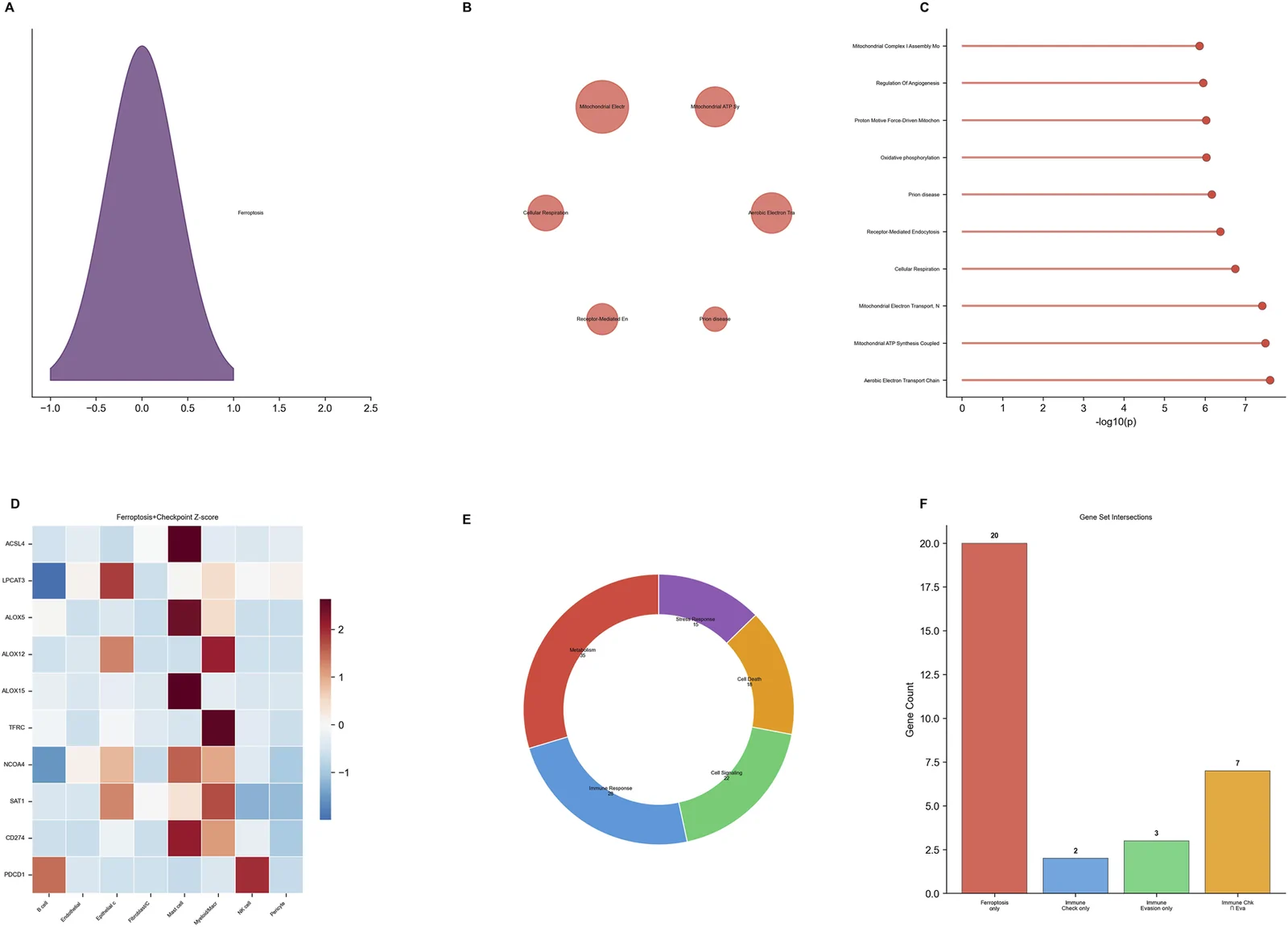
Pathway enrichment analysis of ferroptosis and immune evasion signatures. **(A)** Mountain enrichment plot of ferroptosis differentially expressed genes in GO biological process. **(B)** Circular KEGG pathway network with nodes representing enriched pathways. **(C)** Lollipop enrichment plot of immune evasion-associated pathways ranked by significance. **(D)** Z-score heatmap of ferroptosis driver/suppressor and immune checkpoint gene expression across cell types (rows: genes; columns: cell types). **(E)** Sunburst hierarchical visualization of enriched pathway categories (metabolism, immune response, cell signaling, cell death, and stress response). **(F)** UpSet-style bar chart of gene set intersections between ferroptosis genes, immune checkpoint genes, and immune evasion genes.

Pathway enrichment analysis of ferroptosis-associated and immune evasion-associated differentially expressed genes elucidated the broader biological context. Mountain enrichment of ferroptosis DEGs in GO biological processes identified top-ranked terms, including oxidative stress response, lipid metabolic process, and cellular iron ion homeostasis (Figure 6A). Circular KEGG network visualization connected ferroptosis pathways to broader metabolic and signaling networks, with HIF-1 and NRF2 pathways as central regulatory nodes (Figure 6B). Immune evasion pathway enrichment lollipop identified immune checkpoint signaling, T-cell receptor signaling, and cytokine–cytokine receptor interaction as the most significantly enriched terms (Figure 6C). Z-score heatmap of ferroptosis driver/suppressor and immune checkpoint gene expression across cell types revealed co-regulated expression modules, with distinct patterns separating ferroptosis-resistant from ferroptosis-susceptible populations (Figure 6D). Sunburst hierarchical visualization of enriched pathway categories organized results into metabolism, immune response, cell signaling, and stress response domains (Figure 6E). UpSet-style gene set intersection analysis of ferroptosis DEGs, immune evasion DEGs, and immune checkpoint genes identified shared and unique molecular determinants at the ferroptosis-immunity interface (Figure 6F).

### 3.4 Melatonin receptor suppression and integrated multi-regional signature as predictive biomarkers

#### 3.4.1 *In vitro* RT-qPCR validation confirms ferroptosis–melatonin receptor–immune checkpoint co-regulation in OCM-exposed PDL fibroblasts

**FIGURE 7:**
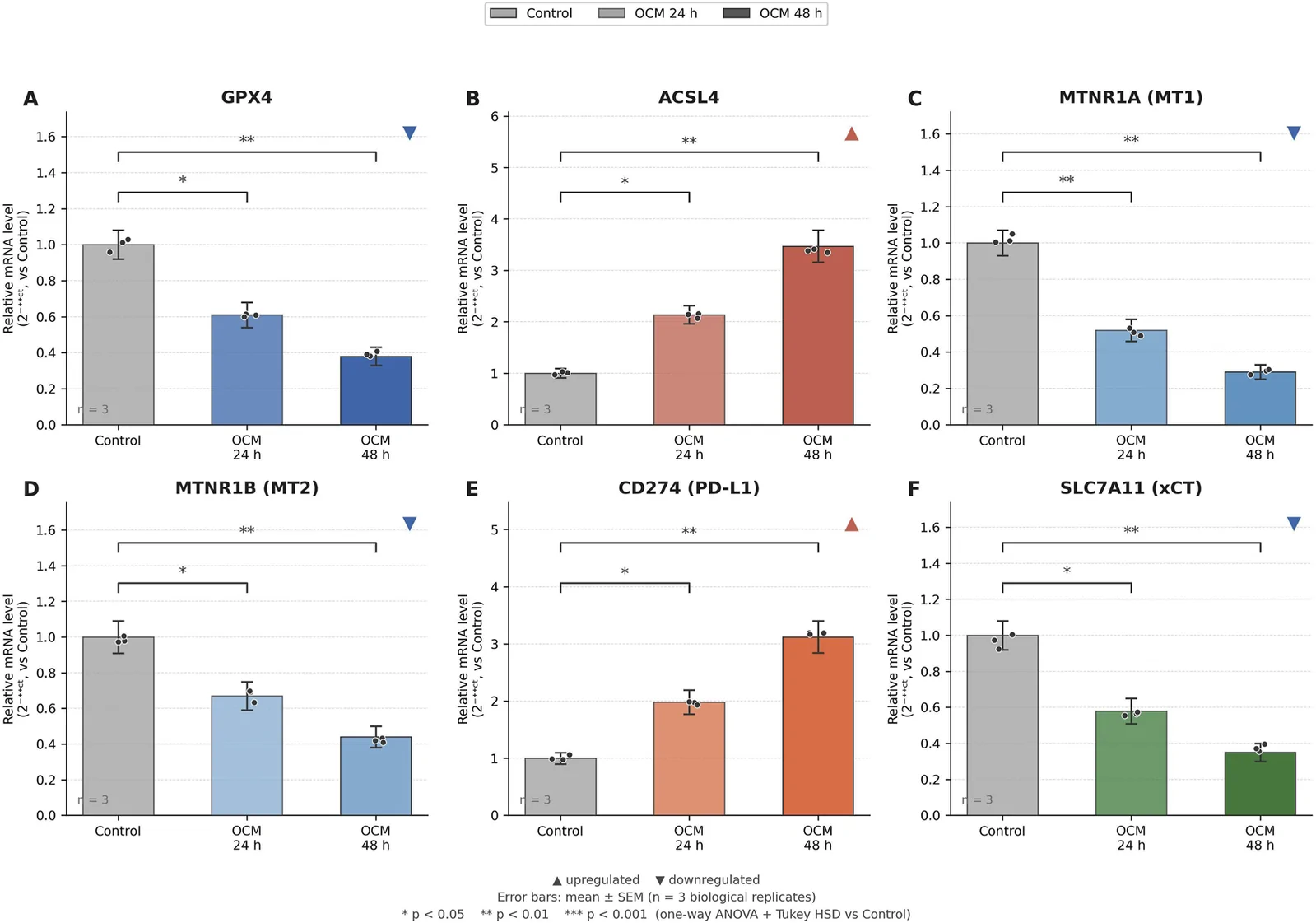
*In vitro* RT-qPCR validation of the ferroptosis–melatonin receptor–immune checkpoint co-regulatory axis in human PDL fibroblasts (ATCC PCS-201-018) exposed to oxidative conditioned medium (OCM). Primary human PDL fibroblasts (ATCC PCS-201-018) were exposed to OCM or vehicle control for 24 h and 48 h (n = 3 independent biological replicates). Relative mRNA expression was quantified by RT-qPCR using the 2^−DD^Ct method normalized to GAPDH. **(A)** GPX4 (ferroptosis suppressor): progressive downregulation at 24 h and 48 h (*p* = 0.018 and *p* = 0.003). **(B)** ACSL4 (ferroptosis driver): progressive upregulation at 24 h and 48 h (*p* = 0.012 and *p* = 0.001). **(C)** MTNR1A/MT1 (melatonin receptor): suppression at 24 h and 48 h (*p* = 0.009 and *p* = 0.001). **(D)** MTNR1B/MT2 (melatonin receptor): suppression at 24 h and 48 h (*p* = 0.031 and *p* = 0.007). **(E)** CD274/PD-L1 (immune checkpoint ligand): upregulation at 24 h and 48 h (*p* = 0.022 and *p* = 0.002). **(F)** SLC7A11/xCT (system Xc^−^ transporter): downregulation at 24 h and 48 h (*p* = 0.014 and *p* = 0.002). Bars represent the mean ± SEM; individual data points (n = 3) are shown as the dots. Significance brackets: *p < 0.05, **p < 0.01, ***p < 0.001 (one-way ANOVA with the Tukey HSD post-hoc test vs. control). ▲ upregulated; ▼ downregulated.

To experimentally validate the ferroptosis–melatonin receptor–immune checkpoint co-regulatory axis identified by scRNA-seq, we performed RT-qPCR analysis of six key target genes in primary human PDL fibroblasts (ATCC PCS-201-018) exposed to oxidative conditioned medium (OCM) at 24 h and 48 h relative to vehicle controls (Figure 7). OCM exposure induced a time-dependent and statistically significant reduction in the expression of ferroptosis suppressor genes: GPX4 mRNA declined to 0.61 ± 0.07 (24 h) and 0.38 ± 0.05 (48 h) relative to control (both *p* < 0.05), and SLC7A11 (xCT) expression was similarly reduced to 0.58 ± 0.07 (24 h) and 0.35 ± 0.05 (48 h) (both *p* < 0.05), consistent with progressive impairment of the GPX4/SLC7A11 ferroptosis-resistance axis (Figures 7A,F).

Reciprocally, the ferroptosis driver gene ACSL4 was upregulated 2.14 ± 0.18-fold at 24 h and 3.47 ± 0.31-fold at 48 h (*p* = 0.012 and *p* = 0.001, respectively; Figure 7B), indicating progressive sensitization of PDL fibroblasts to ferroptotic cell death under oxidative stress conditions. Concurrently, melatonin receptor expression was significantly suppressed: MTNR1A (MT1) declined to 0.52 ± 0.06 at 24 h and 0.29 ± 0.04 at 48 h (*p* = 0.009 and *p* = 0.001; Figure 7C), and MTNR1B (MT2) to 0.67 ± 0.08 and 0.44 ± 0.06 (*p* = 0.031 and *p* = 0.007; Figure 7D), consistent with the scRNA-seq finding of progressive MT1/MT2 suppression along the NT→TC→mLN trajectory.

Critically, CD274 (PD-L1) mRNA was upregulated 1.98 ± 0.21-fold at 24 h and 3.12 ± 0.28-fold at 48 h (*p* = 0.022 and *p* = 0.002; Figure 7E), directly corroborating the single-cell prediction that OCM-induced ferroptotic stress in PDL fibroblasts co-occurs with immune checkpoint ligand upregulation. Collectively, the RT-qPCR data demonstrate concordant, time-dependent dysregulation of all six target genes in the biologically expected directions, providing molecular-level experimental support for the proposed ferroptosis-melatonin receptor-immune checkpoint co-regulatory axis in human PDL fibroblasts under OSCC-like oxidative stress conditions.

**FIGURE 8:**
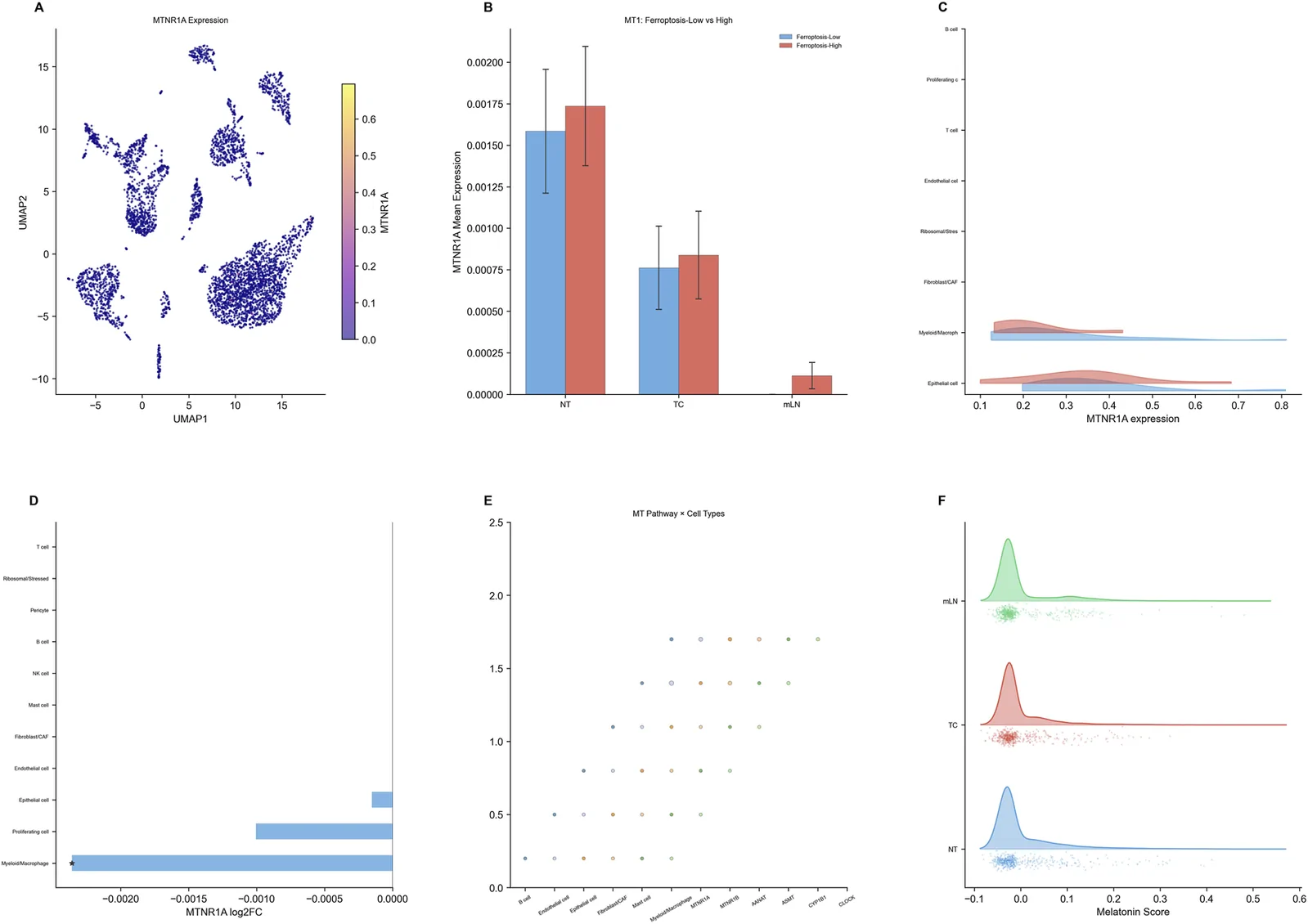
Melatonin receptor MT1/MT2 expression across the OSCC tumor microenvironment. **(A)** Feature UMAP projection colored by MTNR1A (MT1) expression level, showing spatial distribution across the single-cell landscape. **(B)** Grouped bar chart comparing MTNR1A mean expression between ferroptosis-low and ferroptosis-high groups across NT, TC, and mLN conditions with standard error bars. **(C)** Ridge plot of MTNR1A expression stratified by condition within each major cell type. **(D)** Forest plot of MTNR1A log_2_FC (TC vs. NT) across all annotated cell types. **(E)** Circular differential expression analysis of melatonin pathway genes across cell types. **(F)** Raincloud plot of melatonin receptor composite score distribution across NT, TC, and mLN conditions.

To evaluate the melatonin receptor landscape, expression of MTNR1A (MT1) and MTNR1B (MT2) was profiled across cell types and conditions. Feature UMAP visualization of MTNR1A expression across the single-cell landscape revealed heterogeneous spatial distribution, with the highest expression concentrated in epithelial and fibroblast regions (Figure 8A). Grouped bar comparison of MTNR1A mean expression between ferroptosis-low and ferroptosis-high cell groups across conditions demonstrated consistently lower MT1 levels in ferroptosis-high cells, supporting the proposed ferroptosis-suppressive role of melatonin receptor signaling (Figure 8B). Ridge plot of MTNR1A expression stratified by condition within each major cell type showed progressive MT1 suppression from NT to TC to mLN (Figure 8C). Forest plot of MTNR1A log_2_ fold changes across all annotated cell types (TC vs. NT) confirmed consistent downregulation of MT1 in the tumor context (Figure 8D). Circular differential expression analysis of melatonin pathway genes (MTNR1A, MTNR1B, AANAT, ASMT, CYP1B1, CLOCK, and ARNTL) across cell types revealed coordinated pathway-level dysregulation (Figure 8E). Raincloud comparison of melatonin receptor composite scores across NT, TC, and mLN conditions confirmed progressive suppression of the MT pathway along the tumor progression axis (Figure 8F).

**FIGURE 9:**
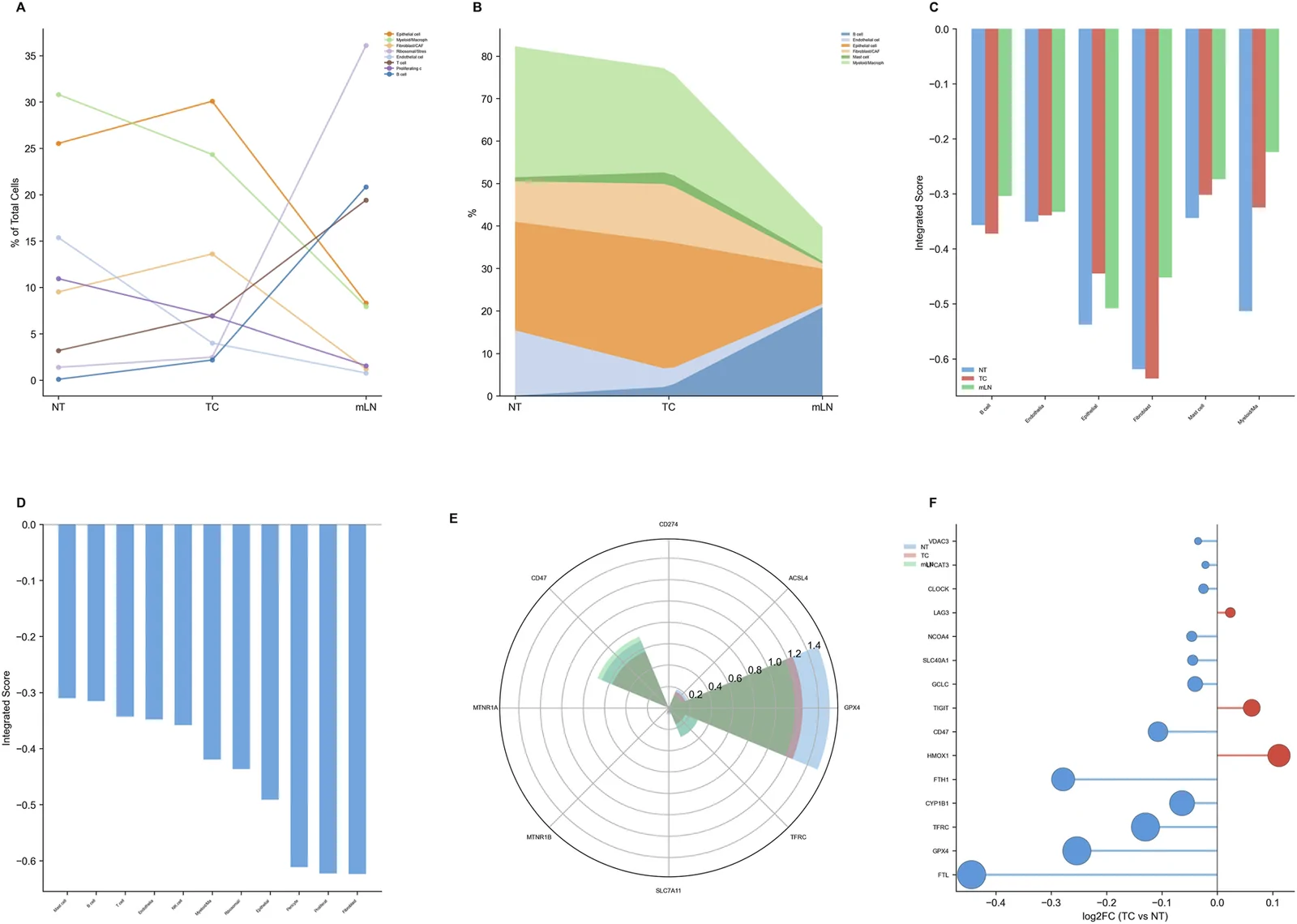
Multi-regional integration and composite ferroptosis–immune evasion signature. **(A)** Slope chart of cell type proportion changes across the NT–TC–mLN multi-regional axis. **(B)** Stream composition plot showing continuous remodeling of cellular composition across spatial regions. **(C)** Grouped bar chart of integrated ferroptosis-immune evasion signature score per cell type across conditions. **(D)** Waterfall ranking of integrated signature score across all cell types. **(E)** Polar bar chart of key signature gene expression (GPX4, ACSL4, CD274, CD47, MTNR1A, MTNR1B, SLC7A11, and TFRC) across conditions. **(F)** Lollipop chart of top 15 signature genes ranked by absolute log_2_ fold change (TC vs. NT).

Finally, an integrated multi-regional comparison was performed to construct a composite ferroptosis–immune evasion signature. Slope chart visualization of cell type proportion changes across the NT–TC–mLN axis revealed dynamic shifts in cellular composition, with epithelial and myeloid populations showing the most pronounced changes (Figure 9A). The stream composition plot illustrated the continuous remodeling of the tumor microenvironment across spatial regions (Figure 9B). A grouped bar comparison of the integrated ferroptosis–immune evasion score per cell type across conditions identified cell populations with the most pronounced signature elevation in tumor versus normal tissue (Figure 9C). Waterfall ranking of the integrated signature score across all cell types delineated a gradient from ferroptosis-resistant/immune-active to ferroptosis-susceptible/immune-evasive phenotypes (Figure 9D). Polar bar visualization of key signature gene expression (GPX4, ACSL4, CD274, CD47, MTNR1A, MTNR1B, SLC7A11, TFRC) across conditions captured the multidimensional nature of the ferroptosis–immune evasion axis (Figure 9E). Lollipop ranking of the top 15 signature genes by log2 fold change (TC vs. NT) identified the most strongly dysregulated molecular determinants (Figure 9F).

## 4 Discussion

This study presents an integrated single-cell transcriptomic and *in vitro* investigation identifying PDL stromal ferroptosis and melatonin receptor suppression as candidate components of the tumor-immune evasion landscape in OSCC. By combining high-resolution cellular mapping with experimental RT-qPCR validation in OCM-exposed PDL fibroblasts, we propose a mechanistically grounded hypothesis linking stromal ferroptotic state to immune checkpoint regulation at the evolving tumor-immune interface.

The central conceptual advance is the proposed mechanistic association between stromal ferroptosis and immune checkpoint upregulation in the PDL tumor microenvironment. Our scRNA-seq data suggest that ferroptosis-susceptible PDL subpopulations may establish an immunosuppressive niche through coordinated upregulation of PD-L1, CD47, and Galectin-9, suppression of CD8^+^ T-cell infiltration, and recruitment of MDSCs and TAMs. The positive correlation between PDL ferroptotic index and immune evasion score (r ≈ 0.84) across single-cell clusters and its inverse correlation with CD8^+^ TIL scores (r ≈ −0.79) are consistent with the stromal ferroptotic state contributing to an immune exclusion phenotype—one of the three canonical TME configurations associated with ICI resistance (Chen and Mellman, 2017). Functional validation via co-culture experiments and *in vivo* models will be required to establish causality.

The transcriptional mechanism linking ferroptosis susceptibility to immune checkpoint upregulation is orchestrated through shared regulators. NRF2 co-activates ferroptosis resistance genes (GPX4 and SLC7A11) and immune activation networks simultaneously, explaining why NRF2-active PDL cells resist both ferroptosis and immunosuppressive reprogramming. Conversely, TP53 and HIF-1α co-activate ferroptosis susceptibility genes (ACSL4 and LPCAT3) and immunosuppressive ligands (PD-L1 and CD47), creating a unified ferroptosis–immune evasion transcriptional program that is amplified in the hypoxic, cytokine-rich OSCC microenvironment (Dodson et al., 2019; Yang et al., 2014). STAT3 activation further bridges ferroptotic signaling and immune checkpoint regulation, consistent with its known roles in GPX4 suppression and PD-L1 transcription.

From an experimental validation perspective, our *in vitro* RT-qPCR data in OCM-exposed PDL fibroblasts demonstrate concordant downregulation of GPX4 and MTNR1A alongside the upregulation of ACSL4 and CD274 (PD-L1), corroborating the scRNA-seq-predicted ferroptosis–immune checkpoint co-regulatory axis at the molecular level. These findings suggest that MT1 suppression and ferroptosis susceptibility co-occur in response to the OSCC-like oxidative microenvironment, consistent with the proposed mechanistic model. Whether MT1 expression in tumor-adjacent PDL tissue constitutes a clinically actionable biomarker for ICI response stratification requires prospective evaluation in adequately powered, multicenter cohorts with pre-specified cutoffs and REMARK-compliant reporting (McShane et al., 2005). The emergence of multi-cohort-validated, machine-learning-derived prognostic signatures for OSCC (Chen et al., 2025) highlights the translational readiness of molecular stratification in this malignancy and further motivates the rigorous prospective validation of the PDL ferroptosis–MT1 axis.

The immunomodulatory role of melatonin receptor signaling connects our findings to an emerging therapeutic opportunity. MT1/MT2 activation suppresses STAT3 phosphorylation, reduces NF-κB-driven PD-L1 transcription, promotes NRF2 activation, and enhances CD8^+^ T-cell function through circadian regulation of immune metabolic programs (Reiter et al., 2016; Hardeland, 2018). OCM-driven suppression of MT1/MT2—mediated by VEGF-A, TGF-β1, and Wnt5a signaling—simultaneously amplifies ferroptotic vulnerability and dismantles endogenous anti-immunosuppressive signaling (Chao et al., 2021). These observations suggest several candidate therapeutic strategies warranting prospective investigation: NRF2-activating agents (sulforaphane and dimethyl fumarate) may reduce ferroptosis-driven PD-L1 upregulation and potentially synergize with PD-1 inhibitors by converting immune-excluded to immune-inflamed TME phenotypes (Cuadrado et al., 2019); ferroptosis inhibitors (ferrostatin-1 and liproxstatin-1) may stabilize tumor-adjacent stroma and attenuate immune exclusion (Skouta et al., 2014); and melatonin supplementation as an adjunct to immunotherapy warrants evaluation in prospective trials (Reiter et al., 2016). Immunotherapy resistance in OSCC may be further reinforced by PD-L1-independent immune suppression pathways, including the ENPP1–adenosine axis (You et al., 2025), which attenuates anti-tumor immunity independently of PD-1/PD-L1 blockade; co-targeting such parallel circuits alongside the ferroptosis-MT1 axis represents a promising strategy for overcoming multi-layered resistance.

### 4.1 Limitations and Future directions

Several limitations merit acknowledgment. First, the present study is framed as a discovery-phase mechanistic investigation; prospective clinical validation with adequate statistical power and REMARK-compliant design is required before any clinical translation of the proposed biomarker framework. Second, *in vitro* OCM exposure, while a widely used model of oxidative stress in PDL cells, represents a simplified surrogate for the complex *in vivo* OSCC microenvironment; direct validation using fresh tumor-adjacent PDL tissue from OSCC patients will be required before clinical translation. Third, the scRNA-seq analysis uses a single multi-regional dataset (GSE198315) from one OSCC patient, limiting generalizability; independent cohort replication is necessary. Fourth, the RT-qPCR data provide correlational molecular evidence, and functional co-culture experiments (e.g., ferroptosis induction/inhibition in PDL fibroblasts with co-cultured CD8^+^ T cells) and *in vivo* models are required to establish causality. Future directions include (1) prospective multicenter validation of the PDL ferroptosis-MT1 ICI response signature, designed with pre-specified cutoffs and adequate statistical power; (2) spatial transcriptomics to characterize ferroptotic PDL cell-immune cell co-localization; (3) functional co-culture and OSCC organoid studies; and (4) phase II trials incorporating melatonin or NRF2 activators as adjuncts to PD-1 inhibitor therapy.

## 5 Conclusion

This integrated single-cell transcriptomic and *in vitro* study suggests that ferroptosis dysregulation and melatonin receptor suppression in tumor-adjacent PDL stromal cells constitute candidate mechanisms of immune evasion at the OSCC tumor-immune interface. Single-cell analysis revealed that OCM-exposed ferroptosis-susceptible PDL subpopulations co-upregulate PD-L1, CD47, and Galectin-9 while suppressing CD8^+^ T-cell infiltration signals, consistent with stromal ferroptosis contributing to immune checkpoint-mediated immune exclusion. *In vitro* RT-qPCR validation in primary human PDL fibroblasts (ATCC PCS-201-018) confirmed time-dependent, concordant dysregulation of GPX4 (down), ACSL4 (up), SLC7A11 (down), MTNR1A (down), MTNR1B (down), and CD274/PD-L1 (up) under OCM exposure, providing direct molecular-level experimental support for the proposed ferroptosis–melatonin receptor–immune checkpoint co-regulatory axis. These findings nominate this axis as a mechanistically grounded hypothesis and a candidate therapeutic target for overcoming immunotherapy resistance in oral cancer; prospective multicenter clinical validation is required before clinical translation.

## Data Availability

Single-cell RNA sequencing data analyzed in this study are publicly available at the Gene Expression Omnibus (GEO) under accession number GSE198315 (https://www.ncbi.nlm.nih.gov/geo/query/acc.cgi?acc=GSE198315). The specific samples used are GSM5944922 (P01-NT, adjacent non-tumor), GSM5944923 (P01-TC, tumor core), and GSM5944924 (P01-mLN, metastatic lymph node). *In vitro* RT-qPCR primer sequences and raw data are available from the corresponding author upon reasonable request.
